# Identification of conserved residues essential for the ciliogenic functions of WDPCP

**DOI:** 10.1242/dmm.052149

**Published:** 2025-11-21

**Authors:** Yeon Ja Choi, Sungbo Hwang, Chanjae Lee, Huiqing Zeng, Xi Chen, Ukhyun Jo, Hyungjin Kim, Aimin Liu, Daeui Park, John B. Wallingford, Jiang Chen

**Affiliations:** ^1^ Department of Pathology, Stony Brook University, Stony Brook, NY 11794, USA; ^2^ Department of Pharmacy, College of Pharmacy, Jeju National University, Jeju 63243, Republic of Korea; ^3^ Center for Biomimetic Research, Division of Advanced Predictive Research, Korea Institute of Toxicology, Daejeon 34114, Republic of Korea; ^4^ Department of Molecular Biosciences, University of Texas at Austin, Austin, TX 78712, USA; ^5^ Department of Biology, Pennsylvania State University, University Park, PA 16802, USA; ^6^ Department of Dermatology, Stony Brook University, Stony Brook, NY 11794, USA; ^7^ Department of Pharmacological Sciences, Stony Brook University, Stony Brook, NY 11794, USA

**Keywords:** *Wdpcp*, Cilia, Hedgehog signaling, Mutation, Structure

## Abstract

Here, we report a genetically engineered mouse model expressing a mutant *Wdpcp* gene that harbors a deletion of two codons encoding D481 and W482 that correspond to N512 and W513 in human WDPCP. Homozygous mutant mice, designated as *Wdpcp*-Z11, exhibited severe developmental abnormalities, including neural tube defects, craniofacial malformation, anophthalmia and polydactyly. The mutant WDPCP protein was expressed but failed to dock to the apical surface of the cell. Cilia formation and Hh signaling were severely impaired. Structure predictions located these residues at the juncture of two alpha helices in a conserved, but otherwise uncharacterized, region of WDPCP. Their absence was predicted to impair the linker and reduce conformational stability of WDPCP. Rescue experiments demonstrated that restoring both D481 and W482 are required for a phenotypic recovery. Because a variant of W513 (p.Trp513Ser) is associated with Bardet-Beidl syndrome, insight gained into the structure-function relationship may be valuable for understanding WDPCP-associated ciliopathy.

## Teaser

**Summary:** A mutation was introduced to a poorly characterized region of mouse WDPCP. Severe ciliogenic defects were associated with comprised structural integrity of a linker between two alpha helices.

## INTRODUCTION

WDPCP, also known as BBS15 and CPLANE5, is a WD-repeat protein that exerts tissue-specific PCP and ciliogenic functions critical for embryonic development of vertebrate animals (Adler and Wallingford, 2017; Kim et al., 2010). WDPCP is a member of the ciliogenesis and planar polarity effector (CPLANE) complex (Toriyama et al., 2016). It forms a complex with INTU, FUZ, RSG1 (also known as CPLANE2) and JBTS17 (also known as CPLANE1) during ciliogenesis, controlling actin-dependent apical migration and docking of basal bodies and the recruitment of intraflagellar transport (IFT)-A proteins to the base of the cilium (Toriyama et al., 2016). These observations suggested that WDPCP is essential for the biogenesis of cilia through forming a functional CPLANE complex that interacts with the IFT proteins.

In mammals, WDPCP plays prominent roles in cilia formation and Hedgehog (Hh) signaling. *Wdpcp*-deficient mouse embryos exhibit cilia-dependent developmental defects such as facial cleft and anophthalmia, polydactyly and cysts in multiple organs (Cui et al., 2013; Toriyama et al., 2016). In humans, several pathogenic sequence variants in *WDPCP* have been reported in ciliopathy patients, including Meckel-Gruber syndrome, Bardet-Biedl syndrome (BBS) and orofaciodigital syndromes (Kim et al., 2010; Saari et al., 2015; Shamseldin et al., 2020; Toriyama et al., 2016). Interestingly, most of these variants are recessive missense mutations. Several of them were mapped to the region in which WDPCP interfaces with INTU; some were linked to interaction between WDPCP and lipids (Langousis et al., 2022). Many other disease-associated alleles remain categorized as variants of unknown functions. Thus, the structure-function relationship in WDPCP remains poorly understood.

WDPCP contains evolutionarily conserved WD40 domains. Using WD40-repeat protein structure predictor (WDSP) and cryogenic electron microscopy (cryo-EM), six WD40 repeats were identified in fly and seven in human WDPCP (Langousis et al., 2022; Wang et al., 2017). These domain analyses provided insights into molecular functions of WDPCP, but the functional importance of these domains remains unclear. Intriguingly, many ciliopathy mutations were located outside of the WD40 domains, warranting further exploration of the structures of WDPCP.

In this study, we generated a mouse model harboring a two-codon deletion in the *Wdpcp* gene. This mutation is located in a highly conserved but poorly defined region distally from the WD40 repeats. It is associated with near complete abrogation of the ciliogenic function of WDPCP. Data generated from this study provide new insight into the structure-function relationship of WDPCP, which is likely to be instrumental in understanding the pathogenesis of WDPCP-associated ciliopathies.

## RESULTS

### ZFN-mediated disruption of *Wdpcp* in mice

**Fig. 1.**
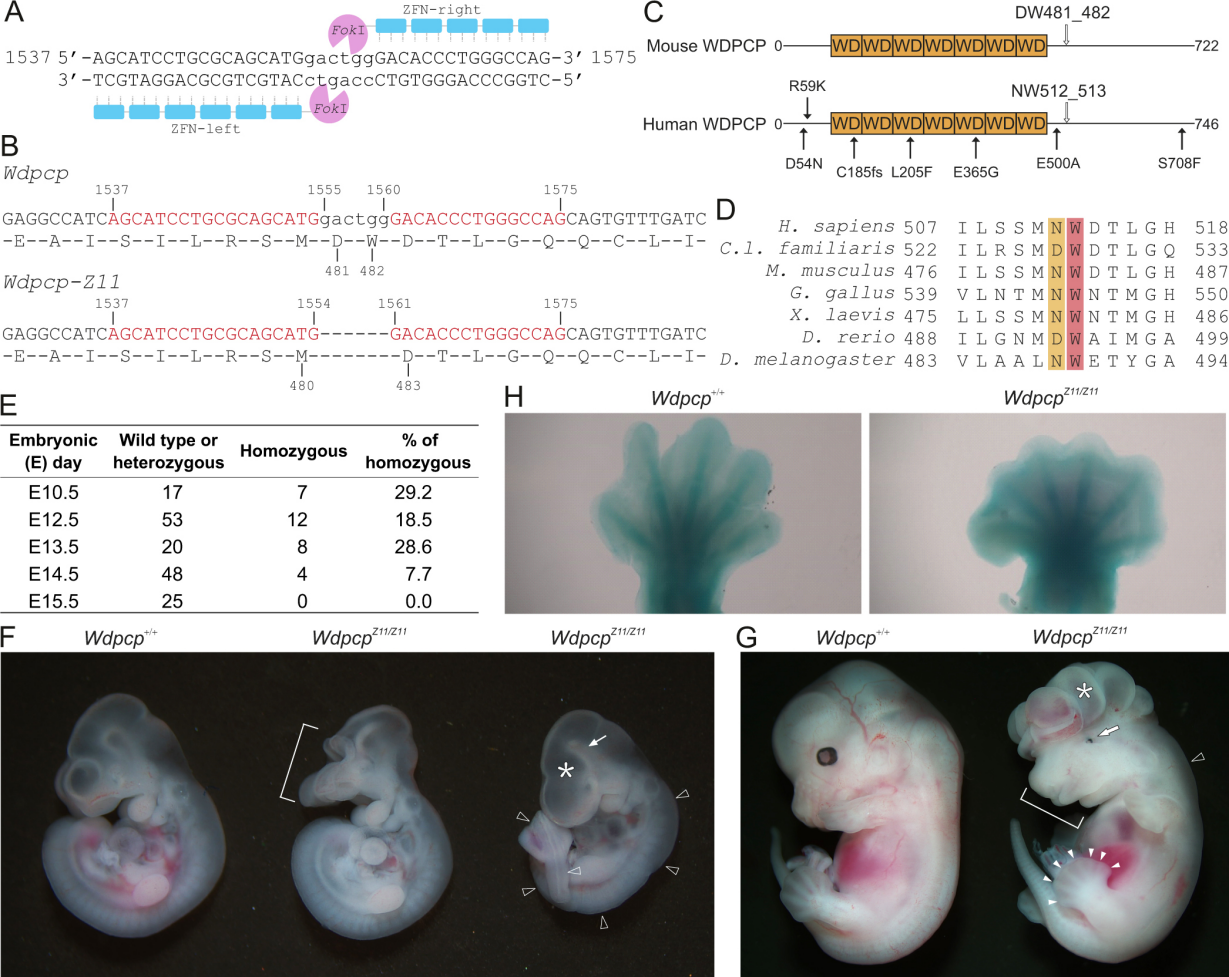
**Generation of a missense mutant *Wdpcp* mouse model.** (A) Illustration of the binding region of a pair of Zinc-finger nucleases (ZFNs) in the mouse *Wdpcp* gene. Nucleotides are numbered according to transcript NM_145425.3. (B) Nucleotide and peptide sequences of wild-type and mutant *Wdpcp* (*Wdpcp-Z11*). Codon 481 and 482 encoding aspartic acid (D) and tryptophan (W), respectively, are absent in *Wdpcp-Z11*. (C) The mouse and human WDPCP peptides and a few known variants. In-frame deletion of aspartic acid (D) in codon 481 and tryptophan (W) 482 in codon 482 (DW481_482) in mouse WDPCP and the corresponding location in human WDPCP (NW512_513) is indicated. (D) Alignment of peptide sequences of WDPCP in selected animal species. Reference sequences (NCBI) are as follows: NP_056994.3, XP_022280509.1, NP_663400.2, XP_015133583.2, XP_018117623.1, XP_005158592.1, NP_001259879.1. (E) Number of embryos generated from heterozygous mating pairs (*Wdpcp^+/Z11^*). (F) Phenotypes of wild-type (*Wdpcp^+/+^*) and homozygous (*Wdpcp^Z11/Z11^*) littermates at embryonic day (E)10.5. Bracket outlines craniofacial defects; asterisk indicates underdeveloped telencephalon; arrow points to tight mesencephalic flexus; open arrowheads indicate twisted body. (G) Phenotypes *Wdpcp^+/+^* and *Wdpcp^Z11/Z11^* at E12.5. Bracket outlines craniofacial anomality; asterisk indicates exencephaly; arrow indicates anophthalmia; open arrowhead points to edema; filled arrowheads indicate polydactyly. (H) Alcian Blue staining of paws from E14.5 *Wdpcp^+/+^* and *Wdpcp^Z11/Z11^* embryos.

A pair of Zinc finger nucleases (ZFNs), comprising two DNA-binding zinc finger proteins fused with the nuclease domain of *Fok*I endonuclease, was engineered (Fig. 1A). The left ZFN (ZFN-left) binds to the mouse *Wdpcp* gene locus in exon 11 that corresponds to cDNA c.1537_1554; the right ZFN (ZFN-right) binds to c.1561_1575. These ZFNs are expected to form a heterodimer and create a double-strand break (DSB) between nucleotides 1555 and 1560 (Fig. 1B).

mRNAs encoding these ZFNs were electroporated into mouse zygotes. CEL I assay identified chimeras, which were then backcrossed to C57BL6/J mice to generate founders. Sanger sequencing of genomic DNA identified that Founder 11 contained an in-frame deletion of six nucleotides (cDNA 1555_1560), which encode aspartic acid (D) and tryptophan (W) at codon 481 and 482 (WDPCP-D481_W482del or WDPCP-ΔDW) (Fig. 1B). This allele was designated as *Wdpcp-Z11*. To mitigate potential off-target events, this allele was backcrossed to C57BL6/J for three generations.

D481 and W482 corresponds to N512 and W513 of human WDPCP, respectively (Fig. 1C). They are located distally from the WD40 repeats (Langousis et al., 2022) (Fig. 1C) in a highly conserved region (Fig. 1D). Interestingly, a variant of unknown function, reported in ClinVar [*NM_015910.7 (WDPCP):c.1538G>C*], was associated with the ciliopathy BBS (rs2104900470). It results in a tryptophan to serine mutation at codon 513 (p.W513S). Thus, the *Wdpcp-Z11* mutant mouse model reported herein is a valuable *in vivo* tool to understand this ciliopathy gene.

### *Wdpcp-Z11* impairs mouse embryonic development

Heterozygous *Wdpcp-Z11* (*Wdpcp^+/Z11^*) mice were phenotypically indistinguishable from wild-type (*Wdpcp^+/+^*) littermates. Homozygous *Wdpcp-Z11* (*Wdpcp^Z11/Z11^*) embryos obtained from *Wdpcp^+/Z11^* mating pairs were unable to survive to embryonic day (E)14.5 (Fig. 1E). *Wdpcp^Z11/Z11^* embryos appeared smaller than *Wdpcp^+/+^* and *Wdpcp^+/Z11^* littermates and exhibited developmental abnormalities, including signs of underdevelopment of the telencephalon, tight mesencephalic flexure, exencephaly, anophthalmia, polydactyly and edema (Fig. 1F-H). Severe neural tube defects might have contributed to embryonic lethality. Because these phenotypes were consistently observed in knockout mouse models of all CPLANE genes, including *Wdpcp* (Cui et al., 2013; Gray et al., 2009; Heydeck and Liu, 2011; Toriyama et al., 2016; Zeng et al., 2010), we conclude that codon 481_482 encoded by c.1555_1560 is essential for *Wdpcp* function in mice.

### *Wdpcp-Z11* impairs Hh signaling

**Fig. 2.**
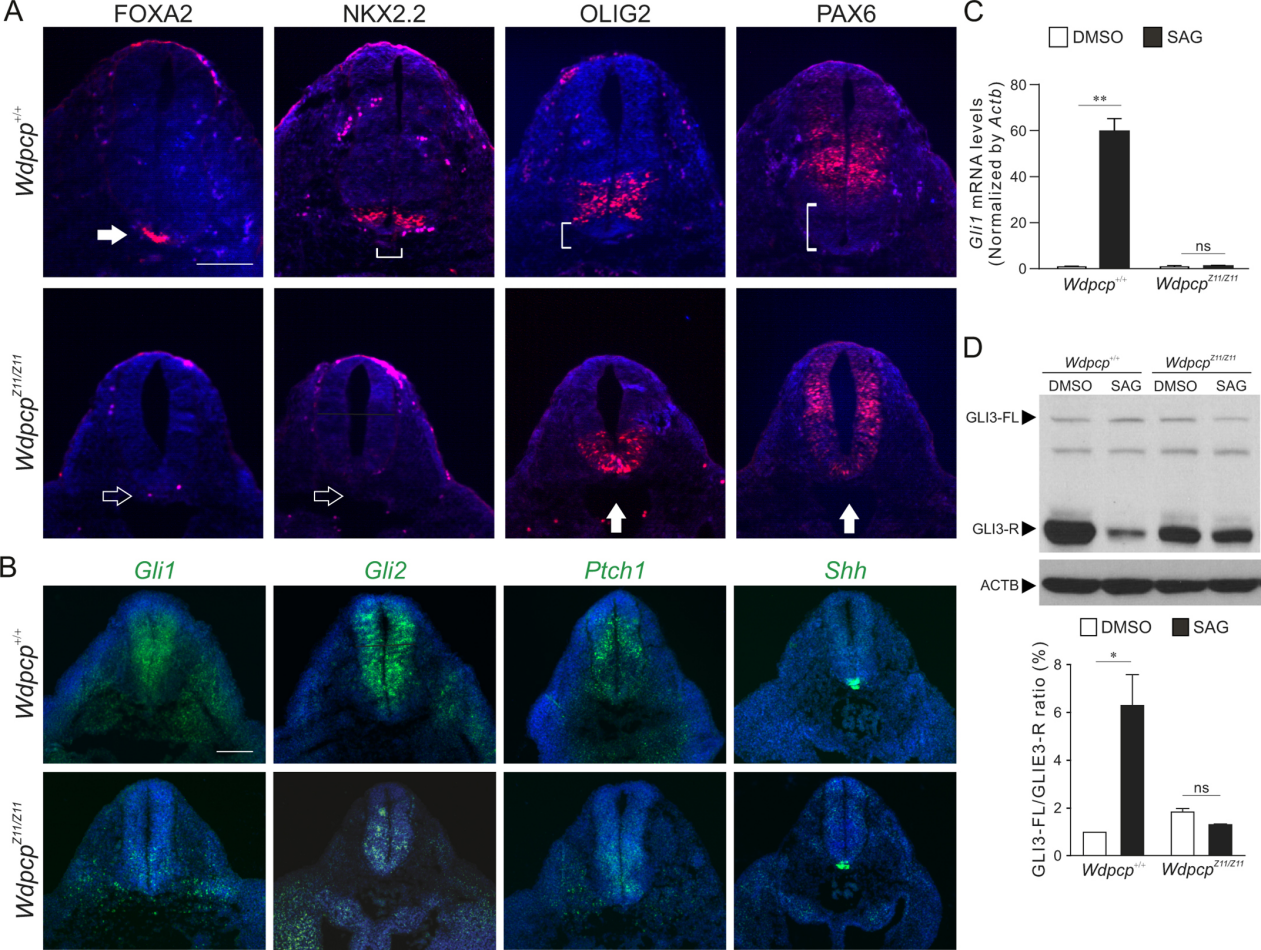
**Impaired Hh signaling in homozygous *Wdpcp* mutant mice.** (A) Immunofluorescence of markers of neural tube patterning in E10.5 wild-type (*Wdpcp^+/+^*) and homozygous (*Wdpcp^Z11/Z11^*) embryos. *n*=3 pairs. Brackets indicate ventral neural tube regions that are devoid of the expression of corresponding markers. Open arrows indicate the lack of the corresponding markers in the ventral region of the neural tube. Filled arrows point to the ventral-most regions of the neural tube that are positive for FOXA2, OLIG2 or PAX6, respectively. (B) *In situ* hybridization of components of the Hh signaling pathway in the neural tube of E10.5 *Wdpcp^+/+^* and *Wdpcp^Z11/Z11^* embryos. *n*=3 pairs. (C) Quantitative RT-PCR of *Gli1* and *Ptch1* in mouse embryonic fibroblasts (MEFs) isolated from *Wdpcp^+/+^* and *Wdpcp^−/−^* embryos and treated with solvent (DMSO) and smoothened agonist (SAG). Experiments were conducted three times. (D) Western blotting of full-length GLI3 (GLI3-FL) and the repressor form of GLI3 (GLI3-R) in cells described in C, and quantification of the ratio between GLI3-FL and GLI3-R. Experiments were repeated three times. ns, not significant; *P<0.05, **P<0.01 (one-way ANOVA). Scale bars: 100 µm.

*Wdpcp* is essential for cilia-mediated Hh signaling (Cui et al., 2013; Kim et al., 2010). The effects of the *Wdpcp-Z11* mutation on Hh-mediated patterning of the developing neural tube was examined. Neural progenitor specification was examined by immunofluorescence of the neural tube of E10.5 embryos between the forelimb and hindlimb in *Wdpcp^Z11/Z11^*. FOXA2 and NKX2.2 diminished in the floorplate and p3 progenitors, respectively, whereas the OLIG2 and PAX6 domains expanded ventrally (Fig. 2A). The Hh pathway was examined by *in situ* hybridization. In *Wdpcp^Z11/Z11^* mutants, *Gli1*, *Gli2* and *Ptch1* were attenuated in the dorsal region of the neural tube (Fig. 2B). The expression pattern of *Shh* did not appear to be significantly affected (Fig. 2B). These results indicated impairment of Hh signaling during the patterning of the neural tube.

Mouse embryonic fibroblasts (MEFs) isolated from wild-type and *Wdpcp^Z11/Z11^* embryos were treated with smoothened agonist (SAG) to examine their responsiveness to ligand-induced Hh pathway activation. Quantitative RT-PCR demonstrated that SAG was able to elicit robust induction of a Hh pathway target gene, *Gli1*, in wild-type MEFs (Fig. 2C). In contrast, *Gli1* transcription did not respond to SAG in *Wdpcp^Z11/Z11^* MEFs (Fig. 2C).

The processing of full-length GLI3 (GLI3-FL) was examined by western blotting. SAG treatment suppressed the processing of GLI3-FL to GLI3 repressor (GLI3-R) in wild-type MEFs, resulting in a significant 6.3±1.9-fold reduction in GLI3-R as determined by the ratio of GLI3-FL to GLI3-R (Fig. 2D). In contrast, the processing of GLI3-FL was severely impaired in *Wdpcp^Z11/Z11^* MEFs because SAG could no longer significantly reduce the processing of GLI3-FL to GLI3-R (Fig. 2D). Data obtained from these experiments demonstrate an essential role of D481 and W482 in Hh signaling.

### *Wdpcp-Z11* impairs ciliogenesis

**Fig. 3.**
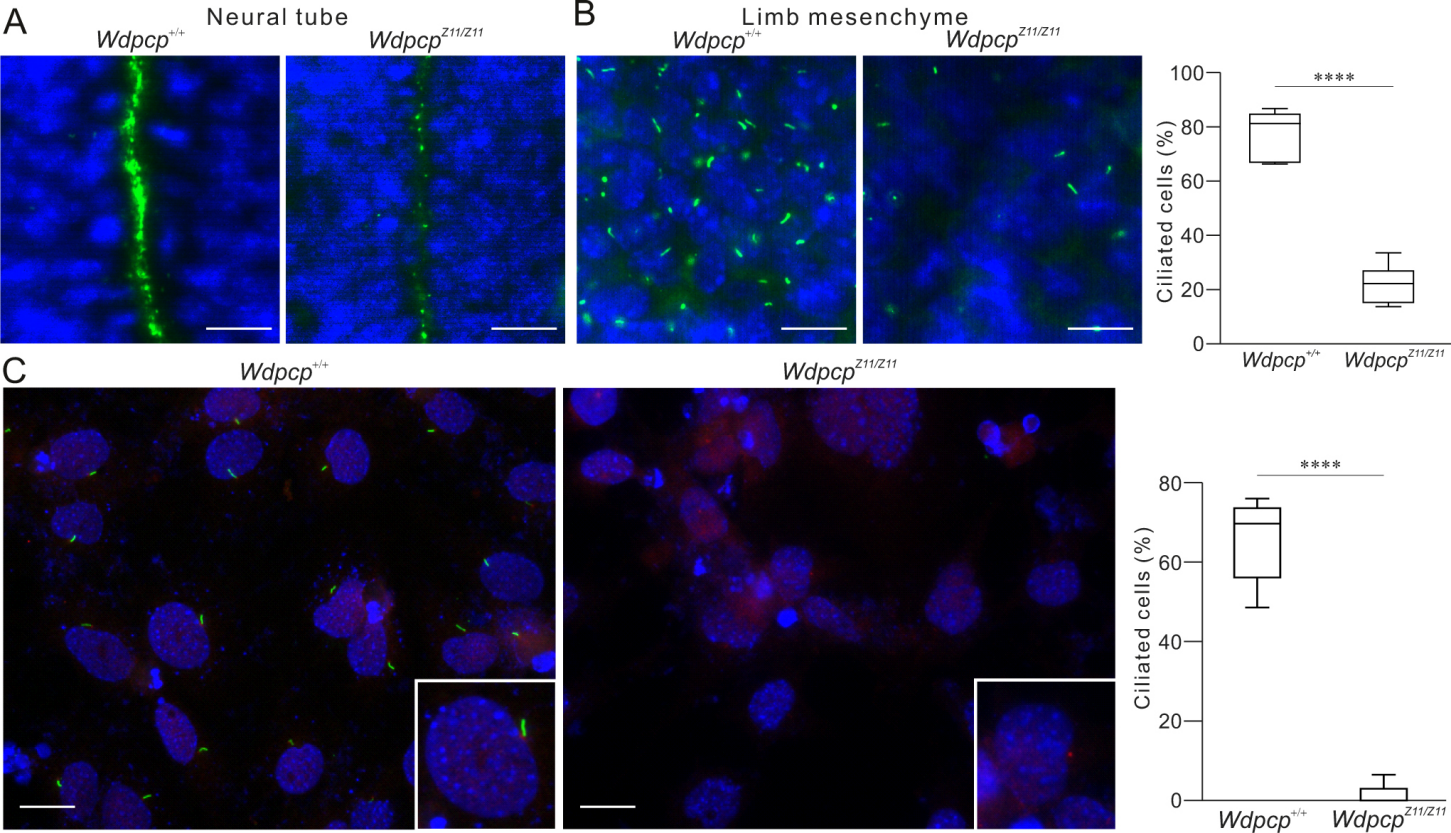
**Impaired primary cilia formation.** (A,B) Immunofluorescence of cilia (ARL13B, green) in neural tube (A) and limb mesenchyme (B) and quantification of ciliated limb mesenchymal cells (B) in E10.5 wild-type (*Wdpcp^+/+^*) and homozygous (*Wdpcp^Z11/Z11^*) embryos. *n*=3 pairs. (C) Immunofluorescence of cilia in MEFs isolated from *Wdpcp^+/+^* and *Wdpcp^Z11/Z11^* embryos and quantification of ciliated cells. Experiments were repeated three times. In the box and whiskers plots, the first quartile, median and third quartile are indicated by the box; the minimum and maximum values are indicated by the whiskers. ****P<0.0001 (unpaired two-tailed Student's *t*-test). Scale bars: 10 µm.

One of the most prominent functions of WDPCP in vertebrates is to facilitate cilia formation. Primary cilia formation in *Wdpcp-Z11* embryos was examined. Immunofluorescence labeling of cilia with ARL13B in E12.5 embryos demonstrated that the neural tube of wild-type embryos was abundantly ciliated, whereas cilia in *Wdpcp^Z11/Z11^* littermates were sparse (Fig. 3A). In the limb mesenchyme, 77.8±9.1% cells were ciliated in wild type, whereas 22.0±7.3% cells were ciliated in *Wdpcp^Z11/Z11^* (Fig. 3B).

MEFs were isolated from wild-type and *Wdpcp^Z11/Z11^* embryos and examined for primarily cilia formation. Wild-type MEFs contained 65.7±10.7% ciliated cells. *Wdpcp^Z11/Z11^* MEFs were essentially unable to form primary cilia, with merely 1.3±3.0% ciliated cells (Fig. 3C). These data suggest that D481 and W482 are critical for the ciliogenic function of *Wdpcp*.

### Mutant WDPCP fails to localize to the basal bodies

**Fig. 4.**
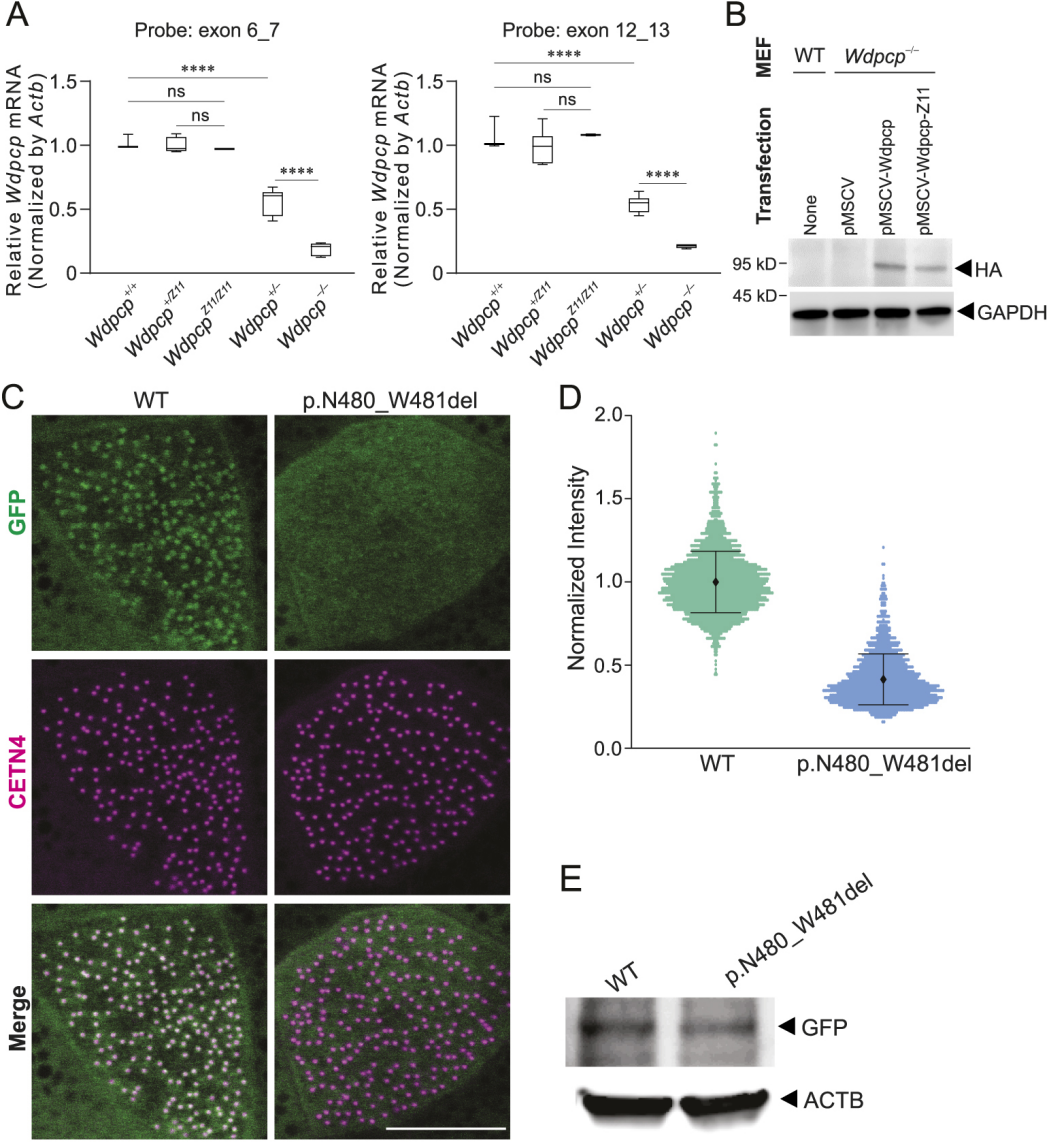
**Expressivity of mutant *Wdpcp*.** (A) Quantitative RT-PCR of mRNA isolated from *Wdpcp^+/+^*, heterozygous and homozygous *Wdpcp-Z11* (*Wdpcp^+/Z11^* and *Wdpcp^Z11/Z11^*), and *Wdpcp* knockout (*Wdpcp^+/−^* and *Wdpcp^−/−^*) embryos. TaqMan probes spanning exon 6_7 and exon 12_13 of mouse *Wdpcp* were used. Experiments were repeated three times. In the box and whisker plots, the first quartile, median and third quartile are indicated by the box; the minimum and maximum values are indicated by the whiskers. ns, not significant; ****P<0.0001 (unpaired two-tailed Student's *t*-test). (B) Western blotting of HA for the WDPCP-HA fusion protein in wild-type (WT) mouse embryonic fibroblasts (MEFs) and *Wdpcp^−/−^* MEFs transfected with empty vector (pMSCV), wild-type *Wdpcp* expressing vector (pMSCV-Wdpcp) or *Wdpcp-Z11* expressing vector (pMSCV-Wdpcp-Z11). Experiments were repeated three times. (C) Immunofluorescence of GFP and centrin 4 (CETN4) in *Xenopus* multiciliated epithelial cells transfected with WT and mutant (p.N480_W481del) Wdpcp-GFP fusion constructs. Scale bar: 10 µm. (D) Quantification of GFP intensity normalized that of CETN4. Experiments were repeated three times. (E) Western blotting of GFP in cells described in C.

To determine whether mutant *Wdpcp-Z11* mRNA was stable, quantitative RT-PCR was performed with TaqMan probes that span the junctions of exon 6_7 and exon 12_13. These probes are located respectively upstream and downstream of the ZFN-targeting site in exon 11. Results consistently demonstrated that the *Wdpcp* transcripts in *Wdpcp^+/Z11^* and *Wdpcp^Z11/Z11^* embryos were expressed at levels comparable to those in the wild type (Fig. 4A). These data demonstrated the stability of the *Wdpcp*-*Z11* transcripts.

To determine whether the WDPCP-ΔDW protein encoded by the *Wdpcp-Z11* allele can be expressed, full-length *Wdpcp* cDNA from the wild-type and *Wdpcp^Z11/Z11^* embryos was cloned into the mammalian expression plasmid MSCV-N-Flag-HA-GFP. These constructs, pMSCV*-Wdpcp* or pMSCV*-Wdpcp-Z11*, were then transfected in MEFs isolated from *Wdpcp* knockout (*Wdpcp^−/−^*) embryos (Toriyama et al., 2016). Western blotting with an anti-HA antibody detected the WDPCP-HA and WDPCP-Z11-HA fusion proteins (Fig. 4B), indicating that the mutant WDPCP-D481_W482del protein was expressed and stable. Antibodies suitable for detecting WDPCP protein in mouse embryos were unavailable. Mass spectrometry was used to detect WDPCP-D481_W482del. Specifically, peptides in MEFs isolated from *Wdpcp-Z11* embryos were digested with trypsin and analyzed by liquid chromatography-tandem mass spectrometry (LC-MS/MS). Mutant WDPCP peptide SMDTLGQQCLIGMGTIVNHLLR was detected in both +2 and +3 charge states with similar LC retention times (Fig. S1).

We next sought to understand the mechanism by which the deletion elicits these effects in mouse embryos. CPLANE proteins are hierarchically recruited to basal bodies in advance of ciliogenesis (Toriyama et al., 2016), so we asked whether the Z11 deletion impacts WDPCP protein localization. To this end, we made the cognate deletion in *Xenopus* Wdpcp, fused this to GFP, and expressed it in multiciliated cells, an effective system for understanding ciliary protein localization (Tu et al., 2018). As expected (Kim et al., 2010), wild-type Wdpcp localized strongly to basal bodies, as quantified by normalizing GFP fluorescence levels against CETN4-RFP (Fig. 4C,D). By contrast, WDPCP-N480_W481del-GFP was not localized at basal bodies and instead was present diffusely throughout the cytoplasm (Fig. 4C,D). Western blots confirmed in *Xenopus* that the WDPCP-N480_W481del protein was stable (Fig. 4E). These data suggest that the Z11 deletion elicits ciliopathic phenotypes as a result of defective localization to the basal bodies.

### Structure and conformational instability of WDPCP-Z11

**Fig. 5.**
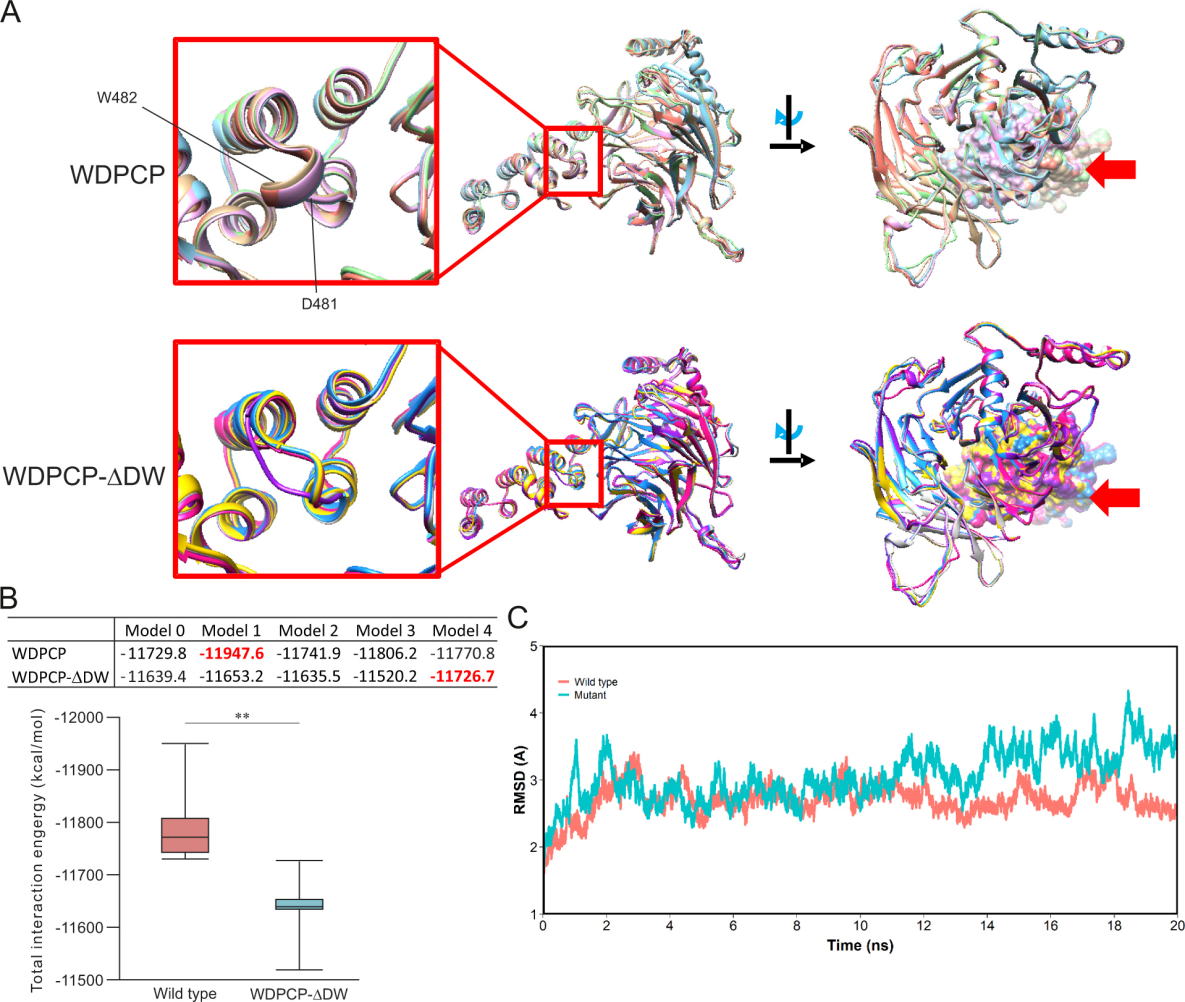
**Structure analysis of mutant WDPCP.** (A) Predicted structures of wild-type mouse (WDPCP) and mutant WDPCP-ΔDW. (B) Total interaction energy distributions. In the box and whisker plots, the first quartile, median and third quartile are indicated by the box; the minimum and maximum values are indicated by the whiskers. **P<0.01 (unpaired two-tailed Student's *t*-test). (C) Plot of root-mean-square deviation (RMSD) in Å, calculated using AR backbone atoms of the wild type and WDPCP-Z11 (mutant) during molecular dynamic simulations. ns, nanoseconds.

To gain insight into the structure-function significance of the D481 and W482 residues, wild-type and mutant mouse WDPCP proteins were modeled by AlphaFold2 (Jumper et al., 2021). The overlap of wild-type and mutant WDPCP proteins using five predictive models is shown in Fig. 5A. The overall structure of mouse WDPCP predicted in the current study includes a seven-bladed beta propeller and several alpha helices at the C-terminus. It was predicted that W482 is located at the start of a small alpha helix loop comprising four residues through L485, whereas D481 is located at the flexible hinge leading to this loop (Fig. 5A). This loop is located in the linker region between two alpha helices. These data were consistent with cryo-EM results (Langousis et al., 2022). Deleting D481_W482 did not appear to change the overall structure of WDPCP (Fig. 5A). However, it disrupted the small helix linker (Fig. 5A). A similar change was predicted in human WDPCP-N512_W513del (Fig. S2).

Based on five models (Model 0 to Model 4), the total interaction energy distributions, calculated by fragment molecular orbital (FMO) were −11,799±79 kcal/mol and −11,635±66 kcal/mol for wild type and mutant, respectively (Fig. 5B, *P*=0.006609), indicating that the disruption of the linker resulted in a less stable structure.

Molecular dynamics predicted conformational change of WDPCP structures with minimum total interaction energy. Model 1 and model 4 were used for analyzing wild-type and mutant WDPCP, respectively (Fig. S3). Average root-mean-square deviation (RMSD) of wild type was 2.709±0.245 Å, while that of WDPCP-Z11 was 3.057±0.380 Å (Fig. 4E, *P*<2.22×10^−16^). The plot of RMSD of trajectories of Cα atoms of WDPCP-ΔDW fluctuated more than that of wild type (Fig. 5C). Wild type exhibited a maximal change in RMSD of 3.410 Å, while WDPCP-ΔDW exhibited a change of 4.331 Å. Also, at 20 ns, RMSDs were 2.539 Å for WDPCP and 3.503 Å for WDPCP-ΔDW (Fig. 5C). This result showed that the structural instability of WDPCP-ΔDW is increasing with time, thereby suggesting that D481 and W482 are critical for conformational stability of WDPCP.

### D481 and W482 are required for the ciliogenic function of *Wdpcp*

**Fig. 6.**
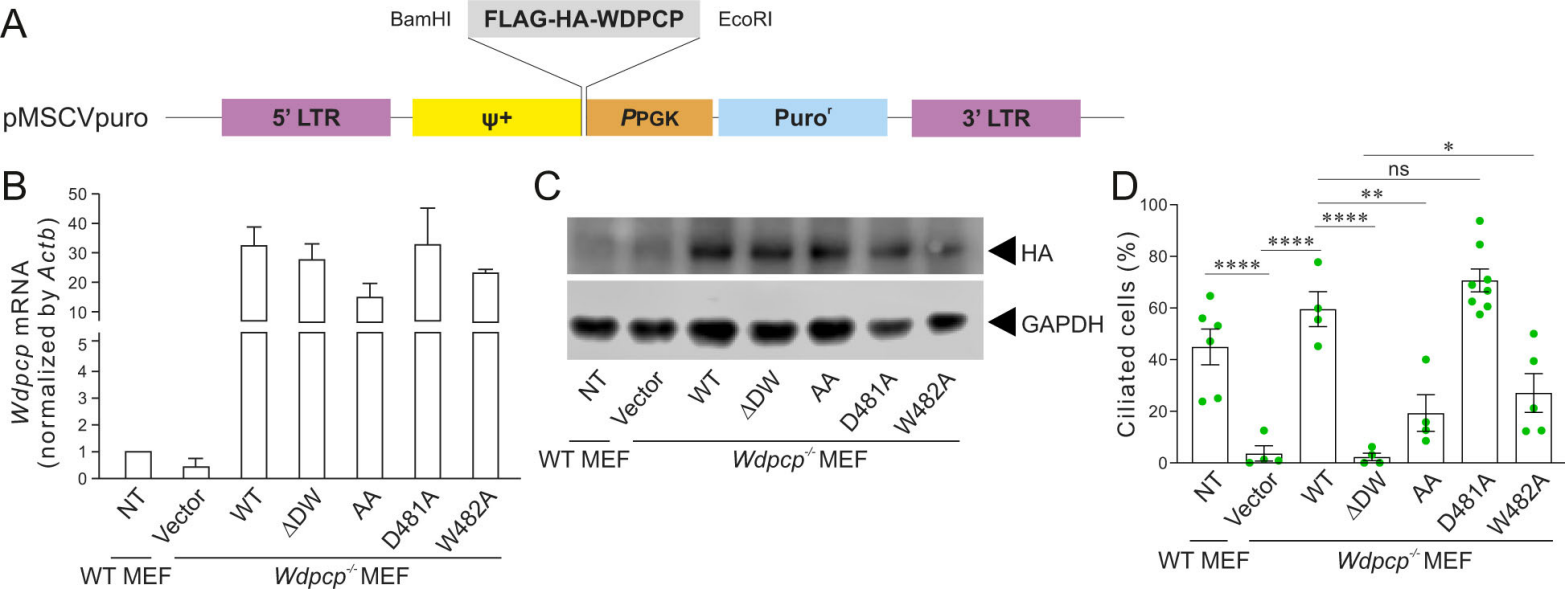
**Rescue of primary cilia formation by wild-type and mutant WDPCP.** (A) Structure of retroviral vector pMSCVpuro used to express WDPCP. (B) Quantification RT-PCR of *Wdpcp* in MEFs isolated from wild-type (*Wdpcp^+/+^*) or null *Wdpcp* (*Wdpcp^−/−^*) embryos that were stably infected with retrovirus expressing wild-type or mutant *Wdpcp* cDNAs. (C) Western blotting of HA in cells described in B. (D) Quantification of ciliated cells in cells described in B. NT, no transfection. ns, not significant; *P<0.05, **P<0.01, ****P<0.0001 (unpaired two-tailed Student's *t*-test).

To experimentally validate these results and gain further insight into functional importance of individual residues, we generated mutant *Wdpcp* expression constructs (Fig. 6A) in which D481 and/or W482 were mutated to alanine simultaneously [*Wdpcp-DW481_482AA* (*Wdpcp-AA*)] or individually (*Wdpcp-D481A* and *Wdpcp-W482A*). Wild type and DW481_482del (ΔDW) were used as controls. These mutant constructs were then used in rescue experiments by transfecting into immortalized *Wdpcp^−/−^* MEFs. First, quantitative RT-PCR and western blotting demonstrated that mutant proteins were stably expressed (Fig. 6B,C). Subsequently, cilia formation was examined. *Wdpcp^−/−^* MEFs transfected with vector (*pMSCV*), as a negative control, were rarely ciliated (3.66±5.92%) (Fig. 6D; Fig. S4). Transfecting *Wdpcp^−/−^* MEFs with *pMSCV-Wdpcp* that encodes wild-type *Wdpcp* (*Wdpcp-WT*) was able to restore ciliogenesis to 59.59±13.60%, a level comparable to that of wild-type MEFs (44.96±16.91%) (Fig. 6D; Fig. S4). As expected, the constructs encoding *Wdpcp-ΔDW* and *Wdpcp-AA* were also unable to achieve significant rescue (2.32±2.96% and 19.27±14.14%, respectively) in comparison to *Wdpcp-WT*. Remarkably, the *Wdpcp-D481A* construct achieved full rescue of ciliogenesis (70.72±12.45%) in the *Wdpcp^−/−^* MEFs, whereas the *Wdpcp-W482A* construct, in which the highly conserved tryptophan residue was mutated to alanine, was unable to rescue the ciliogenesis (27.09±16.91%) in *Wdpcp^−/−^* MEFs in comparison to *Wdpcp-WT* (Fig. 6D; Fig. S4). Data obtained from this experiment suggested that both D481 and W482 are important for the ciliogenic functions of WDPCP, but W482 appeared to be more crucial.

### D481 and W482 are essential for Hh signaling

**Fig. 7.**
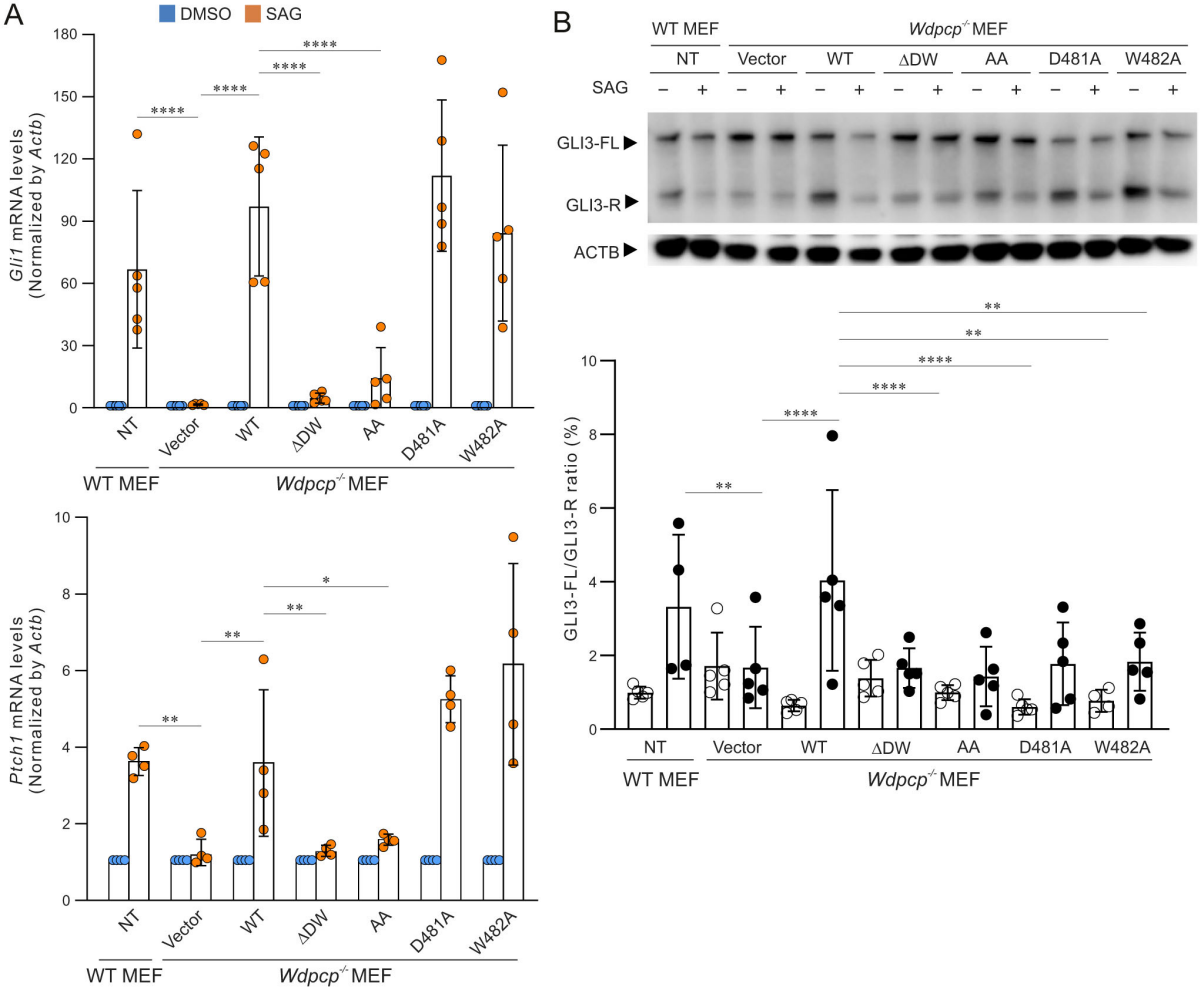
**Rescue of Hh signaling by wild-type and mutant WDPCP.** (A) Quantification RT-PCR of *Gli1* and *Ptch1* in MEFs isolated from wild-type (*Wdpcp^+/+^*) or null *Wdpcp* (*Wdpcp^−/−^*) embryos that were stably infected with retrovirus expressing wild-type or mutant *Wdpcp* cDNAs. (B) Western blotting of full-length GLI3 (GLI3-FL) and the repressor form of GLI3 (GLI3-R) in cells described in A and quantification of the ratio between GLI3-FL and GLI3-R. Experiments were repeated three times. *P<0.05, **P<0.01, ****P<0.0001 (unpaired two-tailed Student's *t*-test).

To determine the significance of D481 and W482 in Hh signaling, *Wdpcp* constructs were transfected into *Wdpcp^−/−^* MEFs, which were then stimulated with SAG. As expected, with SAG, *Wdpcp-WT* rescued the Hh signaling, as quantitative RT-PCR demonstrated marked inductions of *Gli1* and *Ptch1* transcriptions in *Wdpcp^−/−^* MEFs (Fig. 7A). In contrast, the *Wdpcp-ΔDW* and *Wdpcp-AA* constructs were unable to induce significant inductions of *Gli1* and *Ptch1* transcription in response to SAG, whereas the *Wdpcp-D481A* and *Wdpcp-W482A* constructs achieved full rescue similar to that with *Wdpcp-WT* (Fig. 7A).

Western blotting was performed to evaluate the processing of GLI3. *Wdpcp-WT* restored the capability of processing of GLI3-FL to GLI3-R in *Wdpcp^−/−^* MEFs such that the ratio of GLI3-FL/GLI3-R was significantly increased in response to SAG treatment in comparison to that in vector-transfected controls (Fig. 7B). Consistent with prior findings, the *Wdpcp-ΔDW* and *Wdpcp-AA* constructs were unable to rescue the GLI3 processing defects in *Wdpcp^−/−^* MEFs such that the ratio of GLI3-FL/GLI3-R remained unchanged in SAG-treated *Wdpcp^−/−^* MEFs (Fig. 7B). *Wdpcp-D481A* and *Wdpcp-W482A* were able to marginally restore the processing of GLI3-FL to GLI3-R in SAG-treated *Wdpcp^−/−^* MEFs such that the GLI3-FL/GLI3-R ratio was only slightly improved (Fig. 7B). These data suggested that both D481 and W482 are required for *Wdpcp* to facilitate ligand-induced Hh pathway activation.

## DISCUSSION

Here, we have shown that deletion of two amino acids in a poorly defined region of the CPLANE subunit WDPCP is sufficient to disrupt basal body localization and, in turn, to render the protein unable to support ciliogenesis or Hh signaling. These residues are highly conserved, and one of them is associated with human ciliopathy. Several other missense mutations in *WDPCP* are associated with ciliopathies (Kim et al., 2010; Saari et al., 2015; Seo et al., 2011; Toriyama et al., 2016), and several of these missense mutations (D54N, R55K, L205F, E365G, S708F) do not affect the expression or stability of the mutant WDPCP proteins (Langousis et al., 2022). It is not surprising then that the in-frame deletion of DW481_482 reported herein did not affect *Wdpcp* expression but must somehow elicit a specific effect on WDPCP protein function.

WDPCP is an integral component of CPLANE complex (Adler and Wallingford, 2017), and it comprises a seven-bladed β-propeller at N-terminus and a series of α-helices; these alpha helices largely mediate its interaction with INTU (Langousis et al., 2022). Interestingly, although DW481_482 are located in a flexible linker within these helices, they are positioned on the surface away from INTU, so it seems unlikely the deletion would affect INTU binding. It is also unlikely that this deletion impacts WDPCP binding to phosphoinositides (PIPs), in particular PI(3)P, because this putative binding region is restricted to the C-terminal 60 amino acid region (residue 662-722), away from D481 and W482 (Langousis et al., 2022). Finally, WDPCP also interacts strongly with JBTS17, which is required for WDPCP localization to basal bodies (Toriyama et al., 2016); because the DW481_482 deletion disrupts recruitment of WDPCP to basal bodies (Fig. 4), one attractive possibility is that the deletion disrupts interaction with JBTS17.

The CPLANE complex facilitates ciliogenesis through the interaction with multiple ciliogenic modules, and the deletion may affect these as well. For example, the CPLANE subunits INTU and FUZ form a cognate GEF for the ciliogenic RAB23 (Gerondopoulos et al., 2019; Sanchez-Pulido and Ponting, 2020). It is plausible then that the D481 and W482 region serves as a platform for recruiting ciliogenic molecules to the site of RAB23 activation. Such a possibility is substantiated by the Mon1-Ccz1-Bulli (MCBulli) complex, which is structurally homologous to CPLANE (Herrmann et al., 2023). In MCBulli, Bulli, which plays a peripheral role similar to WDPCP, may participate in recruiting additional regulators of endolysosomal trafficking to sites of RAB7 activation. WDPCP has also been implicated in control of actin dynamics, and this function may be independent of the other CPLANE subunits (Cui et al., 2013; Park et al., 2015). Given the key role of actin dynamics in ciliogenesis (Hufft-Martinez et al., 2024), it is also possible that the DW481_482 deletion exerts its effect via actin.

In sum, the *Wdpcp-Z11* allele reported herein represents a novel mouse model expressing a loss-of-function missense mutant of the human ciliopathy protein WDPCP. It should be a useful tool for investigating ciliopathy and the structure-function relationship of WDPCP and CPLANE.

## MATERIALS AND METHODS

### Generation of mutant *Wdpcp* allele

*Wdpcp* ZFNs were custom designed by the CompZr product line (Sigma-Aldrich, St Louis, MO, USA). ZFNs are engineered proteins comprised of a FokI endonuclease domain and multimeric zinc finger protein motifs capable of binding triplets of DNA sequences (Porteus and Carroll, 2005). The complexity of the CompZr zinc finger motif libraries enables the targeting of most DNA sequences. ZFNs can cleave double-stranded DNA after forming a dimer. The six-finger design of ZFN-left and five finger design of ZFN-right directs these ZFNs to the preselected *Wdpcp* DNA sequences and introduces a site-specific DSB. This break may be repaired by either homology-dependent repair or non-homologous end joining (NHEJ) (Cathomen and Joung, 2008). NHEJ-mediated repair may result in the loss of nucleotides.

Specifically, ZFN-left binds to the antisense strand of the mouse *Wdpcp* genomic DNA (c.1537-1554, numbered according to cDNA sequence); ZFN-right binds to the sense strand of the mouse *Wdpcp* genomic DNA (c.1561-1575), in exon 11. These ZFNs were cloned into the pZFN plasmid (Sigma-Aldrich) and expressed *in vitro*. These ZFN mRNAs were electroporated into early zygotes at approximately four- to eight-cell stage by the Transgenic Core facility of the University of Colorado Denver Anschutz Medical Campus (Aurora, CO, USA). Embryos were subsequently transferred to pseudo-pregnant recipient female mice to generate chimeric offsprings. CEL I assay was used to detect mutations in the *Wdpcp* gene in chimeras. All animal-related experiments were approved by the Institutional Animal Care and Use Committee (IACUC) of the University of Colorado Denver and Stony Brook University.

### MEF isolation and treatment

MEFs were prepared from E12.5 embryos of wild type (*Wdpcp^+/+^*), *Wdpcp^−/−^* (Toriyama et al., 2016) and *Wdpcp^Z11/Z11^*. Briefly, each embryo was placed on dishes, and the head, liver and heart were removed for determining genotypes. The remaining parts of the embryo were washed by sterile PBS and placed in 0.05% trypsin-EDTA, minced and incubated in 0.05% trypsin-EDTA for 20 min at 37°C. The lysate was then passed through a needle (20 G) fitted on a syringe several times to make a single-cell suspension. Dissociated cells were cultured in Dulbecco's modified Eagle medium supplemented with 10% fetal bovine serum and 100 U/ml penicillin/streptomycin. MEFs were immortalized by infecting with pLenti-CMV-SV40 (Addgene plasmid #22298).

For determining ciliogenesis and Hh signaling, MEFs were grown to confluency in complete medium. Medium was replaced with starving medium containing 0.25% serum for 24 h. Then, MEFs were treated with 100 nM SAG (Calbiochem, San Diego, CA, USA) in starving medium for 24 h to activate Hh signaling.

### Plasmid construction, retroviral vector production and generation stable cells

The full-length of mouse WDPCP was amplified by PCR reaction with primers (5′-GGATCCACCATGTCTTTCTGCTTGACTGAA-3′; 5′-GAATTCCACCAAACCAAAGTGAAC-3′) and subcloned into pcDNA3-Flag through the BamHI/EcoRI sites. Point mutations (D481A and W482A) and a deletion mutant of WDPCP-D481_W482del were generated by using the QuikChange II XL Site-Directed Mutagenesis Kit (Agilent Technologies, Santa Clara, CA, USA) and confirmed by DNA sequencing. Mouse *Wdpcp* cDNA was also introduced into a retroviral vector, pMSCV-Flag-HA, through the BamHI/EcoRI sites for establishing stable cell lines expressing WDPCP wild-type and mutants. Retrovirus was produced in HEK293T cells after transfection of pMSCV, packaging and envelope vectors. The medium containing virus particles was harvested twice for 2 days after transfection and used to treat *Wdpcp^−/−^* MEFs after filtration. Stable cell lines were established by selecting infected cells with 2 μg/ml puromycin.

### Immunofluorescence

Freshly isolated embryos were fixed immediately in buffered formalin, embedded in paraffin or subjected to other examinations. Most immunofluorescence labeling of tissue specimens and cells was performed on formalin-fixed paraffin-embedded tissue sections as described previously (Dai et al., 2013; Yang et al., 2015). The following primary antibodies were used: anti-FOXA2 [4C7, Developmental Studies Hybridoma Bank (DSHB), Iowa City, IA, USA, 1:25), anti-NKX2.2 (74.5A5, DSHB, 1:40), anti-OLIG2 (AB9610, Millipore, Piscataway, NJ, USA, 1:1000), anti-PAX6 (Pax6, DSHB, 1:500), anti-γ-TUB (ab11317, Abcam, Cambridge, UK, 1:500) and anti-ARL13B (73-287, NeuroMab, Davis, CA, USA, 1:100). AlexaFluor-conjugated secondary antibodies (1:200) were obtained from Life Technologies (Carlsbad, CA, USA). Sections were sealed in mounting medium with or without DAPI (Vector Laboratories, Burlingame, CA, USA). Images were acquired by a Nikon (Melville, NY, USA) 80*i* fluorescence microscope, fitted with a Nikon DS-Qi1Mc camera, or by a Leica (Wetzlar, Germany) SP5C spectral confocal laser-scanning microscope, and processed with Photoshop 5.5 CS (Adobe System Incorporated, San Jose, CA, USA). Ciliated cells were calculated by dividing the number of cells with a primary cilium with the total number of cells (determined by DAPI) in randomly selected microscopic fields.

### RNA isolation and quantitative RT-PCR

RNA was isolated with the RNeasy kit (Qiagen), and quantitative RT-PCR analyses were performed as described previously (Choi et al., 2019). Briefly, cDNA was synthesized from 1 μg total mRNA using a SuperScript II First-Strand Synthesis System (Invitrogen, Carlsbad, CA, USA) and random hexameric primers. Real-time quantitative RT-PCR was performed on ABI Prism 7500 (Applied Biosystems, Foster City, CA, USA) with the following TaqMan probes (Life Technologies, Grand Island, NY, USA): *Wdpcp*, Mm00520644_m1 and Mm01234287_m1; *Gli1*, Mm00494654_m1, *Ptch1*, Mm00436026_m1; *Actb*, Mm00607939_s1. Results were analyzed using ΔΔCt method. Relative expression levels of target genes were determined by comparing with wild type or treatment controls after normalization with *Actb*.

### Western blotting

Protein was extracted either by homogenizing tissue or cells in cold RIPA lysis buffer (50 mM Tris-HCl pH 7.4, 150 mM NaCl, 1% Triton X-100, 1% sodium deoxycholate and 0.1% SDS) supplemented with proteinase inhibitors. Cell lysates were cleared by centrifugation at 13,000 ***g*** for 20 min at 4°C; protein concentration was determined by a BCA Protein assay Kit (Pierce) and measured using a SpectraMax plate reader (Molecular Devices, San Jose, CA, USA). Equal amounts of protein were loaded on a 10% SDS-PAGE and transferred to Hybond Nitrocellulose (GE Healthcare, Chicago, IL, USA) membranes and probed with the primary antibodies at 4°C overnight. These antibodies were as follows: anti-HA (11867423001, Roche, 1:1000), anti-GLI3 (AF3690, R&D Systems, 1:200), anti-GAPDH (2118S, Cell Signaling Technology, Danvers, MA, USA, 1:1000) and anti-β-actin (sc-47778, Santa Cruz Biotechnology, Dallas, TX, USA, 1:1000). HRP-conjugated secondary antibodies (BD Biosciences, San Jose, CA, USA, 1:250) were then applied for 1 h. SuperSignal substrates (Thermo Fisher Scientific, Waltham, MA, USA) and CL-XPosure film (Thermo Fisher Scientific) were used for detection. Quantification was performed by using densitometry and ImageJ software (National Institute of Health, Bethesda, MA, USA).

### LC-MS/MS

Proteins were extracted from MEFs isolated from *Wdpcp^Z11/Z11^* embryos in 5% SDS, 100 mM triethyl ammonium bicarbonate (TEAB), reduced in 10 mM dithiothreitol and alkylated in 25 mM iodoacetamide. Protein was precipitated by phosphoric acid and S-Trap bind/wash buffer (90% methanol/50 mM TEAB). Samples were then loaded on an S-Trap mini cartridge (K02-mini-10 Protifi, Fairport, NY, USA) and washed with 90% methanol and 100 mM TEAB. The samples were digested with TPCK trypsin (Sigma-Aldrich, 4352157). Peptides were eluted by sequential addition of 80 μl 50 mM TEAB, 0.2% formic acid, 50% acetonitrile and 0.2% formic acid, dried and resuspended in 25 µl 0.1% formic acid. Peptides were analyzed by C18 reverse phase LC-MS/MS.

Peptide identification and quantitation was performed using an orbitrap instrument (Q Exactive HF, Thermo Fisher Scientific) followed by protein database searching. Replicate samples were analyzed, using two different HPLC gradient profiles (0-30% ACN over 90 min and 0-40% ACN over 90 min). Electrospray ionization was conducted at 2.3 kV. Information-dependent MS acquisitions were made using a survey scan covering 375-1400 m/z at 60,000 resolution, followed by the ‘top 20’ consecutive second product ion scans at 15,000 resolution. Data files were acquired with Xcalibur (Thermo Fisher Scientific). Peptide alignments and quantitation were performed using Proteome Discoverer v3.1 software (Thermo Fisher Scientific). The mouse UniProt dataset (2019_04 release) was used for alignment.

### *Xenopus* experiments

Experiments using *Xenopus* were performed protocols approved by IACUC at the University of Texas at Austin (Austin, TX, USA). *Xenopus* embryos manipulations were carried out using standard protocols. The construct of *Xenopus* Wdpcp N480_W481del mutant was generated by performing mutagenesis on a plasmid containing *Xenopus* Wdpcp tagged with GFP, using a QuikChange II Site-Directed Mutagenesis Kit (200523, Agilent Technologies). Capped mRNAs of GFP-WT and mutant WDPCP, as well as RFP-centrin, were synthesized using a mMESSAGE mMACHINE SP6 transcription kit (AM1340, Thermo Fisher Scientific). mRNAs of 100 pg (GFP-WDPCP) and 40 pg (RFP-centrin) were injected into two ventral blastomeres of four-cell-stage embryos. Confocal images were captured with an LSM700 inverted confocal microscope (Carl Zeiss, Dublin, CA, USA) with a Plan-APOCHROMAT 63×/1.4 NA oil immersion objective. Imaging processing was conducted using ImageJ, and graphs were generated with R studio. Western blotting was carried out according to standard protocols. The antibodies used were as follows: anti-GFP (sc-9996, Santa Cruz Biotechnology, 1:200), HRP-conjugated goat anti mouse IgG (H+L) secondary antibody (31430, Thermo Fisher Scientific, 1:5000) and anti-beta-actin (6009-1, Proteintech, 1:20,000).

### WDPCP structure prediction

The 3D structure of mouse WDPCP^Z11^ was predicted by AlphaFold2 (Jumper et al., 2021). The amino acid sequence of mouse WDPCP was downloaded as a FASTA file from UniProt (ID: Q8C456). The amino acid sequence for WDPCP^Z11^ had deletion of Asp481 and Trp482 of the wild-type mouse WDPCP. The complete database of AlphaFold was used for structural predictions and analysis. The maximum template release date was defined as 14 May 2020. Other parameters of AlphaFold2 were set to default. The structures of wild-type WDPCP and WDPCP^Z11^ were generated based on five structural models prepared using AlphaFold2 had hydrogen atoms bound to heavy atoms.

### Fragment molecular orbital (FMO) calculation

To calculate the interaction energy among the residues in WDPCP and WDPCP^Z11^, FMO calculations were performed using the predicted WDPCP and WDPCP^Z11^ structures. FMO-based calculations are reported to yield predicted values having a high correlation with the experimental data (Fedorov and Kitaura, 2007). All FMO calculations in this study were performed using the FMO-DFTB3 method (Gaus et al., 2011). All input files were prepared in compliance with the hybrid orbital projection fragmentation scheme (Nakano et al., 2000). One fragment was defined as two cysteine residues that were within 2.15 Å from each other and were composed of disulfide bonds. The polarizable continuum model was used considering that the binding of the RBDs to the receptors occurs in a solution state (Tomasi et al., 2005). The protein stability for each predicted structure was determined relative to the total interaction energy (TIE). The median TIEs of all predicted structure of WDPCP and WDPCP^Z11^ were compared using Kruskal–Wallis rank sum test to obtain a statistical difference in the median values of PIE between WDPCP and WDPCP^Z11^. FMO calculation was performed using the version 30 June 2020, R1 GAMESS (Barca et al., 2020). Statistical analysis for TIE was performed using R, version 4.1.0. TIE distribution plots were generated using the ggplot2 library in R, version 4.1.0.

### Molecular dynamics

To determine protein stability, molecular dynamics simulations were performed to generate WDPCP and WDPCP^Z11^ structures using NAMD version 2.12 (Phillips et al., 2020). The force field parameter used was the Chemistry at HARvard Macromolecular Mechanics (CHARMM) 36 m force field (Huang and MacKerell, 2013). The files of the predicted complex structures were converted from the Protein Data Bank format (.pdb) to the structure (.psf) and coordinate file (.pdb) formats using CHARMM-GUI (Jo et al., 2008). The water box was prepared using Visual Molecular Dynamics (VMD) version 1.9.4 (Humphrey et al., 1996). The simulation was performed with a constant temperature set at 310 K. Energy minimization for the superimposed structure of the Omicron variant was performed for 500 ps with a time step of 1 fs to achieve steady compliance. After energy minimization, the production step was performed for a period of 20 ns. The RMSDs of the WDPCP and WDPCP^Z11^ structures were defined in terms of their alignment to the initial structures. The hydrogen was not included in the RMSD calculation. The RMSD plot was generated using the ggplot2 library in R, version 4.1.0.

### Statistical analysis

All quantifications are presented as mean±s.d. Unpaired two-tailed Student's *t*-test was used unless otherwise stated. One-way ANOVA was conducted using GraphPad software. *P*<0.05 was considered statistically significant.

## Supplementary Material

10.1242/dmm.052149_sup1 Supplementary information
