# Supplementary material for: Identification of conserved residues essential for the ciliogenic functions of WDPCP
Source: Dis Model Mech. 2025 Nov 21;18(11):dmm052149. doi: 10.1242/dmm.052149 (PMC12673966; doi:10.1242/dmm.052149)
Supplement: Supplementary information [file dmm-18-052149-s1.pdf]

+2 charge

Peptide Summary

Sequence: SMDTLGQQCLIGMGTIVNHLLR, C9-Carbamidomethyl (57.02146 Da), N18-Deamidated (0.98402 Da), S1-Dehydrated (-18.01057 Da), T4-Dehydrated (-18.01057 Da), T15-Dehydrated (-18.01057 Da)
Charge: +2, Monoisotopic m/z: 1202.61743 Da (+17.1 mmu/+14.22 ppm), MH+: 2404.22759 Da, RT: 72.0343 min,
Identified with: Sequest HT (v1.17); XCorr:1.52,
Fragment match tolerance used for search: 0.1 Da
Fragments used for search: y-H_2O; y-NH_3; b; b-H_2O; b-NH_3; y

Fragment Matches

Value Type: Theo. Mass [Da]

Ion Series | Neutral Losses | Precursor Ions | Internal Fragments

#1	b^+	b^{2+}	Seq.	y^+	y^{2+}	#2
1	70.02874	35.51801	S-Dehydra...			22
2	201.06922	101.03825	M	2335.17194	1168.08961	21
3	316.09617	158.55172	D	2204.13145	1102.56937	20
4	399.13328	200.07028	T-Dehydrat...	2089.10451	1045.05589	19
5	512.21734	256.61231	L	2006.06740	1003.53734	18
6	569.23881	285.12304	G	1892.98333	946.99530	17
7	697.29739	349.15233	Q	1835.96187	918.48457	16
8	825.35596	413.18162	Q	1707.90329	854.45528	15
9	985.38661	493.19694	C-Carbami...	1579.84471	790.42600	14
10	1098.47068	549.73898	L	1419.81407	710.41067	13
11	1211.55474	606.28101	I	1306.73000	653.86864	12
12	1268.57620	634.79174	G	1193.64594	597.32661	11
13	1399.61669	700.31198	M	1136.62447	568.81588	10
14	1456.63815	728.82271	G	1005.58399	503.29563	9
15	1539.67527	770.34127	T-Dehydrat...	948.56253	474.78490	8
16	1652.75933	826.88330	I	865.52541	433.26634	7
17	1751.82774	876.41751	V	752.44135	376.72431	6
18	1866.85469	933.93098	N-Deamid...	653.37294	327.19011	5
19	2003.91360	1002.46044	H	538.34599	269.67663	4
20	2116.99766	1059.00247	L	401.28708	201.14718	3
21	2230.08173	1115.54450	L	288.20302	144.60515	2
22			R	175.11895	88.06311	1

Fragment Spectrum

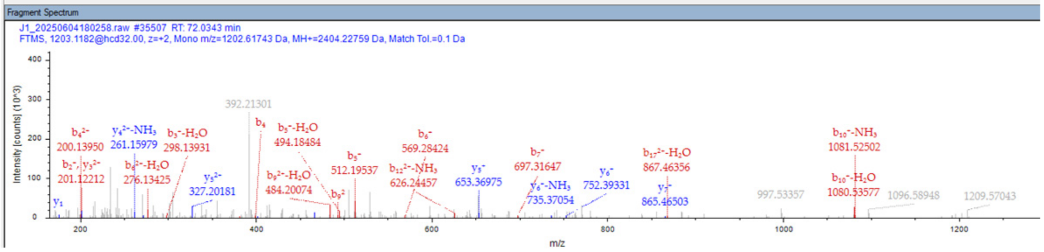

+3 charge

Peptide Summary

Sequence: SMDTLGQQCLIGMGTIVNHLLR, C9-Carbamidomethyl (57.02146 Da), N18-Deamidated (0.98402 Da), S1-Dehydrated (-18.01057 Da), T4-Dehydrated (-18.01057 Da), T15-Dehydrated (-18.01057 Da)
Charge: +3, Monoisotopic m/z: 802.08142 Da (+12.11 mmu/+15.1 ppm), MH+: 2404.22971 Da, RT: 72.0277 min,
Identified with: Sequest HT (v1.17); XCorr:2.43,
Fragment match tolerance used for search: 0.1 Da
Fragments used for search: y-H_2O; y-NH_3; b; b-H_2O; b-NH_3; y

Fragment Matches

Value Type: Theo. Mass [Da]

Ion Series | Neutral Losses | Precursor Ions | Internal Fragments

#1	b^+	b^{2+}	b^{3+}	Seq.	y^+	y^{2+}	y^{3+}	#2
1	70.02874	35.51801	24.01443	S-Dehydra...				22
2	201.06922	101.03825	67.69459	M	2335.17194	1168.08961	779.06216	21
3	316.09617	158.55172	106.03691	D	2204.13145	1102.56937	735.38200	20
4	399.13328	200.07028	133.71594	T-Dehydrat...	2089.10451	1045.05589	697.03969	19
5	512.21734	256.61231	171.41063	L	2006.06740	1003.53734	669.36065	18
6	569.23881	285.12304	190.41779	G	1892.98333	946.99530	631.66596	17
7	697.29739	349.15233	233.10398	Q	1835.96187	918.48457	612.65881	16
8	825.35596	413.18162	275.79017	Q	1707.90329	854.45528	569.97262	15
9	985.38661	493.19694	329.13372	C-Carbami...	1579.84471	790.42600	527.28642	14
10	1098.47068	549.73898	366.82841	L	1419.81407	710.41067	473.94287	13
11	1211.55474	606.28101	404.52310	I	1306.73000	653.86864	436.24819	12
12	1268.57620	634.79174	423.53025	G	1193.64594	597.32661	398.55350	11
13	1399.61669	700.31198	467.21041	M	1136.62447	568.81588	379.54634	10
14	1456.63815	728.82271	486.21757	G	1005.58399	503.29563	335.86618	9
15	1539.67527	770.34127	513.89661	T-Dehydrat...	948.56253	474.78490	316.85903	8
16	1652.75933	826.88330	551.59129	I	865.52541	433.26634	289.17999	7
17	1751.82774	876.41751	584.61410	V	752.44135	376.72431	251.48530	6
18	1866.85469	933.93098	622.95641	N-Deamid...	653.37294	327.19011	218.46250	5
19	2003.91360	1002.46044	668.64272	H	538.34599	269.67663	180.12018	4
20	2116.99766	1059.00247	706.33741	L	401.28708	201.14718	134.43388	3
21	2230.08173	1115.54450	744.03209	L	288.20302	144.60515	96.73919	2
22				R	175.11895	88.06311	59.04450	1

Fragment Spectrum

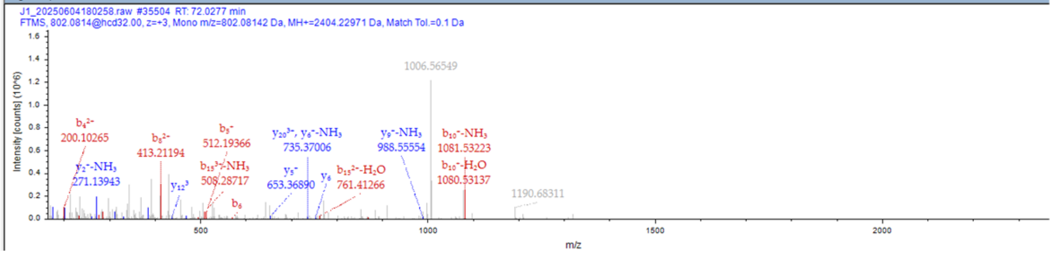

Fig. S1. Mass spectrometry analyses of peptides obtained from MEFs of *Wdpcp-Z11* embryos.

Disease Models & Mechanisms • Supplementary information

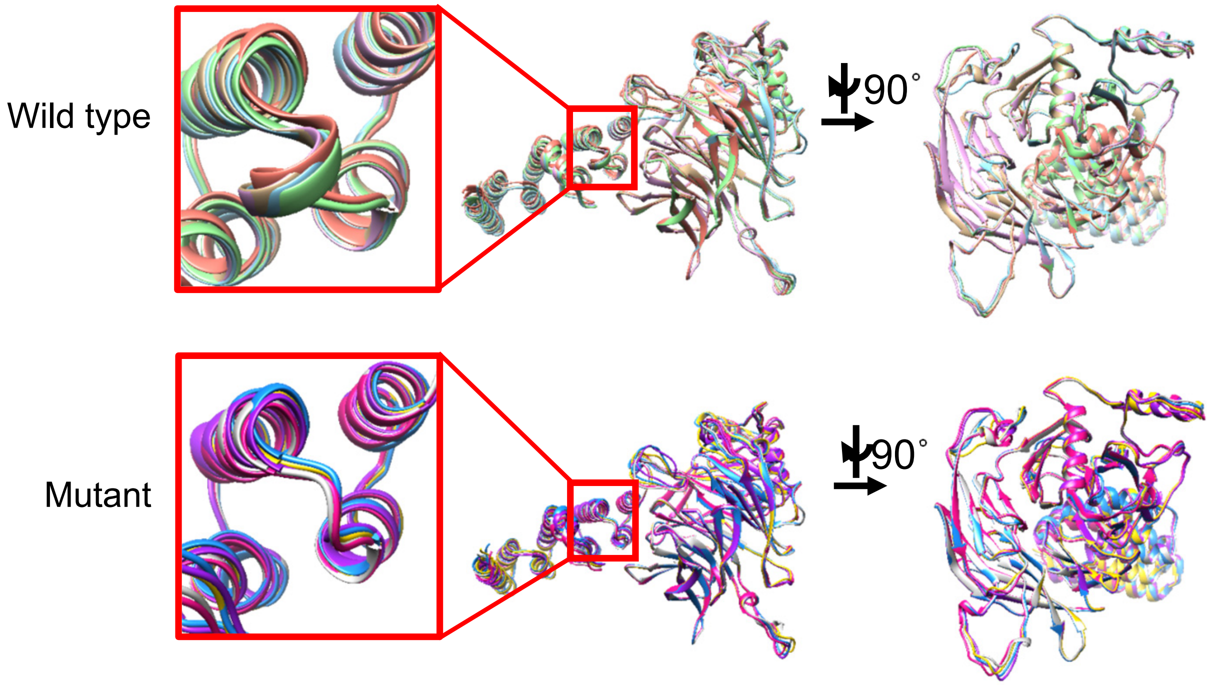

Fig. S2. Predicted structures of human WDPCP (Wild type) and WDPCP-N512_W513del (Mutant).

Disease Models & Mechanisms • Supplementary information

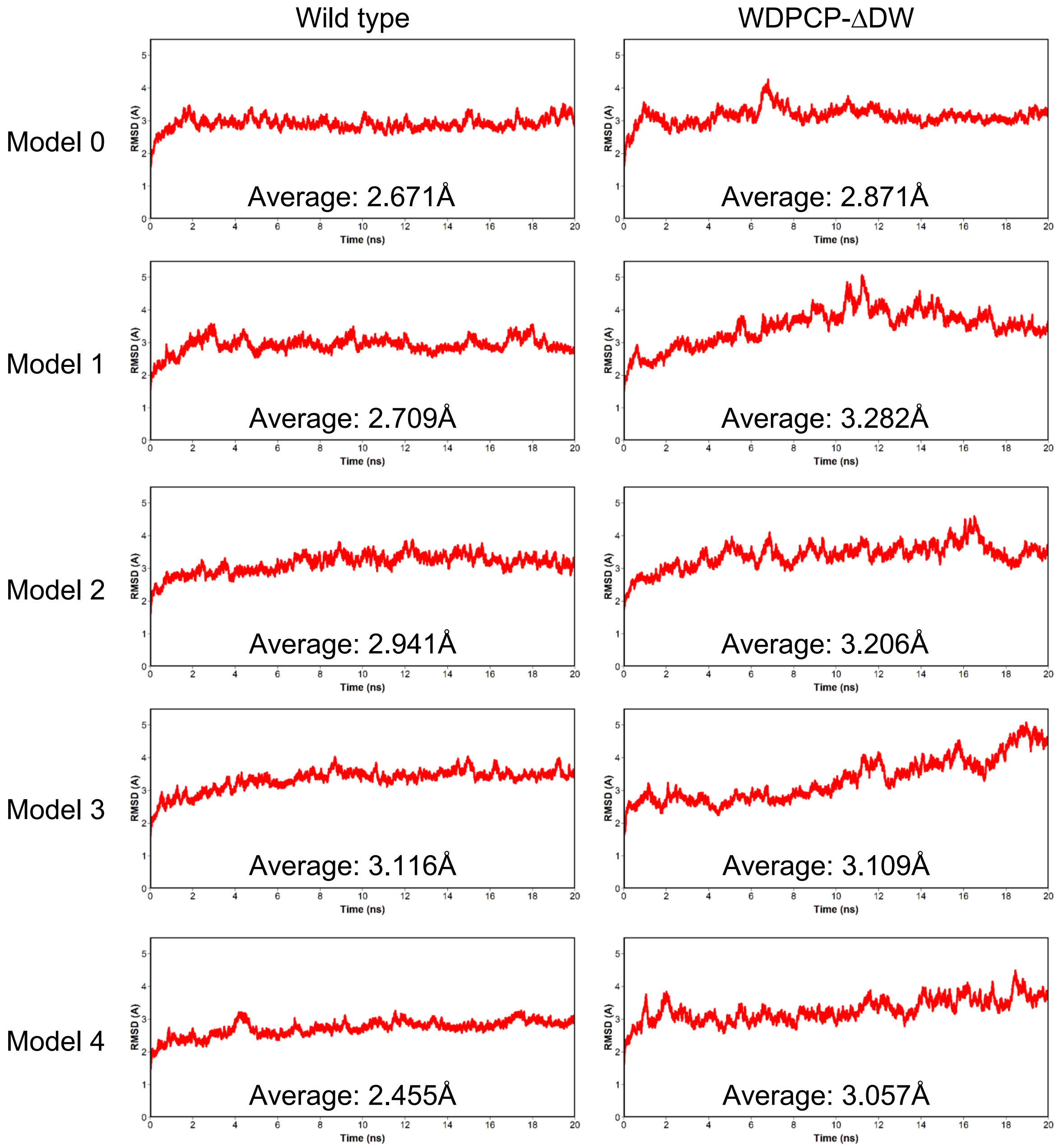

Fig. S3. The average root-mean-square distance (RMSD) of the five models used in molecular dynamics analyses of mouse WDPCP.

Disease Models & Mechanisms • Supplementary information

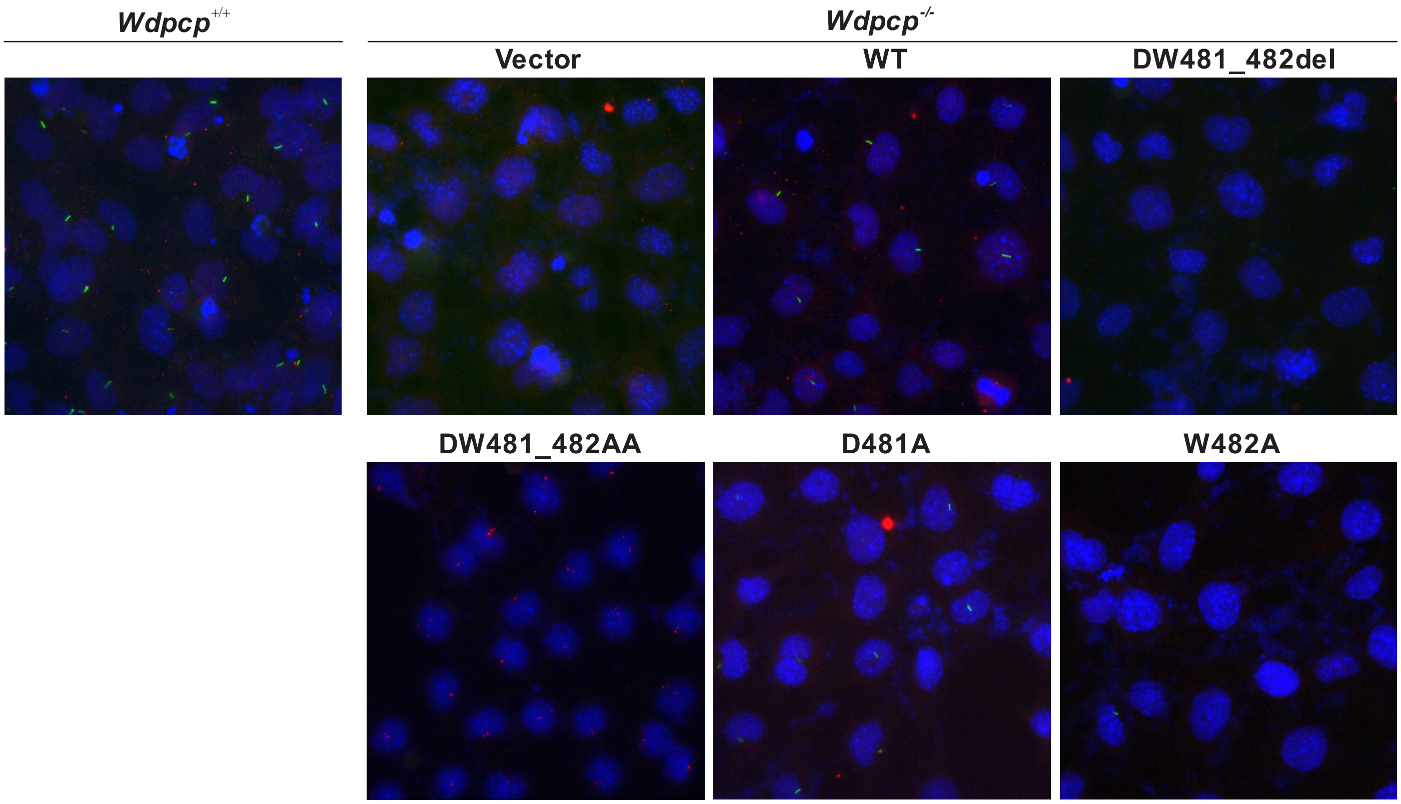

Fig. S4. Immunofluorescence of primary cilia. Primary cilia were labeled with ARL13B (green) in wild type (*Wdpcp*$^{+/+}$) cells or Wdpcp null cells (*Wdpcp*$^{-/-}$) overexpressing lentiviral vectors that expressing wild-type (WT) or mutant *Wdpcp* genes. Images were chosen from three independent experiments.
